# Survey of Persistent Organochlorine Contaminants (PCDD, PCDF, and PCB) in Fish Collected from the Polish Baltic Fishing Areas

**DOI:** 10.1100/2012/973292

**Published:** 2012-04-19

**Authors:** Jadwiga Piskorska-Pliszczynska, Sebastian Maszewski, Malgorzata Warenik-Bany, Szczepan Mikolajczyk, Lukasz Goraj

**Affiliations:** Department of Radiobiology, National Veterinary Research Institute, Partyzantow 57, 24-100 Pulawy, Poland

## Abstract

Concentrations and congener-specific profiles of PCDDs, PCDFs, dl-PCBs, and ndl-PCBs were determined in five species of edible fish from the Baltic Sea (ICES 24–27): salmon (*Salmo salar*), Baltic herring (*Clupea harengus membras*), sprat (*Sprattus sprattus balticus*), sea trout (*Salmo trutta m.trutta*), and cod (*Gadus morhua callarias*). Marker PCBs were the dominant compounds (0.07–60.84 ng/g  w.w.), followed by dl-PCBs (0.64–6.07 pg WHO-TEQ/g w.w.) and PCDD/PCDFs (0.22–5.67 pg WHO-TEQ w.w). The concentration levels of contaminants varied between species. Salmon possessed the highest concentrations (up to 14.11 ± 2.36 pg WHO-TEQ/g  w.w.) and cod the lowest ones (0.84 ± 0.14 pg WHO-TEQ/g  w.w.). Congener profile in the fish tested had similar pattern. The largest contribution to the dioxin toxicity was caused successively by PCB 126, 118, 156, furans (2,3,4,7,8-PeCDF and 2,3,7,8-TCDF), and two dioxins: 1,2,3,7,8-PeCDD and 2,3,7,8-TCDD. Although the dietary consumption of fish from southwest region of the Baltic Sea did not represent a risk for human health (because of very low consumption of marine fish), the excessive eating of some of them may be of significance importance for health of various subgroups of consumers (fishermen).

## 1. Introduction

Organochlorine compounds, such as polychlorinated dibenzo-p-dioxins (PCDDs), polychlorinated dibenzofurans (PCDFs), and polychlorinated biphenyls (PCBs) are pollutants widely distributed in the environment. These compounds have mainly anthropogenic origins. Dioxins (PCDD/PCDFs) are unintended byproducts found in association with certain industrial sites, waste incinerators, and combustion processes, especially of chlorinated material. In human, exposure to dioxin-like PCBs (dL-PCBs) also plays an important role. These are non-*ortho-* and mono-*ortho-* PCBs, 2,3,7,8-TCDD isostereomers that cause biochemical and toxic effects as well through the Ah receptor. Hence, defining the risk of exposure to dioxins, dL-PCBs [1, 2] are included too. As a result of the European Commission strategy to reduce human exposure, dioxins are subjected to mandatory monitoring in food and feed in Member States [3].

These persistent, bioaccumulative organic pollutants can cause long-term impact on wildlife, whole ecosystems, and human health. Long-term exposure to dioxins and PCBs may affect immune response, reproduction functions, and central nervous system and may cause cancer at high exposure levels. These compounds act at the cellular level, disrupting the flow of genetic information as a result of switching on and off some various genes at different time and not in the right way. Marker PCBs (ndl-PCBs), although they act by other mechanisms, are also toxic to humans [2].

Organochlorine contaminants are also common pollutants to the Baltic Sea. Secondary to development of industry and agriculture and increase of the Baltic region population, the Baltic Sea has been seriously contaminated by these toxic chemicals. Pollutants enter the sea from the air or by numerous waterways and become stored in the seabed sediments, where they accumulate throughout the years. In the aquatic food chain, poorly water-soluble dioxins are adsorbed on mineral and organic particles suspended in water, where they are subjected to bioconcentration in trophic chains [4–11]. The ingestion of dioxin-contaminated foods contributes to more than 90% of the total human exposure, with fish and seafood being recognized amongst the main contributors [1]. On the other hand, saltwater fish are an important component of a healthy diet, containing low levels of saturated fats and high levels of polyunsaturated fatty acids beneficial to the prevention of coronary heart disease and also providing other dietary benefits like being a source of valuable protein, vitamins, and minerals (including magnesium, calcium, fluorine, iodine, selenium) [12]. However, fish reservoir harvested from polluted waters may also contain harmful chemicals in concentrations that pose a potential health hazard.

To evaluate the risk of dioxins exposure in the general population and to determine the time trends, regular testing of levels of these compounds in environmental food chain was recommended. European Commission has established random monitoring of dioxins and dL-PCBs in food and feed and defined the action levels, which trigger follow-up investigation to reduce or eliminate the source of contamination (466/2001, 2002/201/EC, 2006/88/EC). Recommendation 2004/705/EC indicates, as guidance, an annual minimum frequency for such monitoring sampling in member states, as well as lays down reporting procedures. Information regarding maximum permit level, sampling, and analysis methods for the official control of dioxins and determination of dioxin-like polychlorinated biphenyls (PCBs) in foodstuffs is laid down in Regulation 1881/2006/EC and 1883/2006/EC. Regulation 882/2004 officially controls and verifies compliance with feed and food law. Protection of human health is one of the fundamental objectives of the food law (178/2002/EC).

European Union's strategy to reduce human dioxins exposure includes mandatory monitoring of food and feed in each member state [3]. The immediate objective of monitoring studies is to obtain information about the levels of contaminants and congener profiles actively identifying potential for reducing human exposure.

This paper reports levels of PCDDs, PCDFs, dioxin-like PCBs, and ndl-PCBs in fish that were collected from the Polish Baltic fishing areas. The study covers the period of official controls from 2006 to 2010, carried out in accordance with the recommendations of the Commission 2004/705/EC and 2006/794/EC [13]. The aim of this study was to determine the concentration levels as well as congener profiles of 35 chlorinated organohalogen compounds.

## 2. Material and Methods

### 2.1. Sampling

 The subjects of the study were several species of Baltic fish collected by Veterinary Inspection in accordance with the recommendations of the Chief Veterinary Officer. CVO recommendations included sampling procedure (selection criteria for sampling), type and size of samples, procedure to be followed in case of exceeding the permissible levels of dioxins, furans, dL-PCBs, or ndl-PCBs and record keeping [14]. National control study included seven 2,3,7,8-congeners of polychlorinated dibenzo-*p*-dioxins, ten 2,3,7,8-congeners of polychlorinated dibenzofurans, twelve dioxin-like PCBs, and six ndl-PCBs. Concentration levels and congener profiles have been studied.

**Figure 1:**
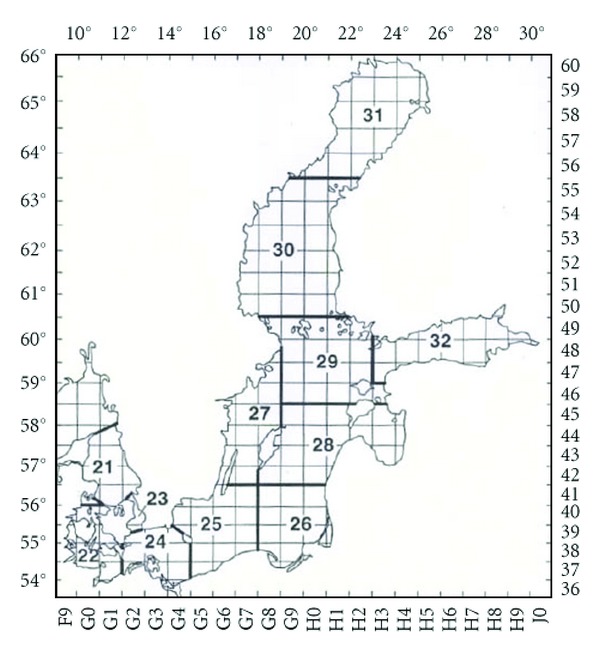
Fishing areas in the Baltic sea. (source: http://www.helcom.fi/environment2/biodiv/fish/en_GB/ICES_subdivisions/).

Baltic fish were sampled by Veterinary Inspection and sent to the National Veterinary Research Institute in Pulawy. Samples were taken from four regions of the Baltic Sea: Baltic West of Bornholm (Subdivision 24), Southern Central Baltic West (Subdivision 25), Southern Central Baltic East (Subdivision 26), and North West of Gotland (Subdivision 27) (Figure 1).

The following fish species were collected: Baltic herring (*Clupea harengus membras*), salmon (*Salmo salar*), sprat (*Sprattus sprattus balticus*), cod (*Gadus morhua callarias*), and sea trout (*Salmo trutta m.trutta*).

### 2.2. Solvents and Standards

 Used solvents and Florisil were obtained from the LGC Standard (Wesel, Germany). Carbopack C and silica gel were from Sigma-Aldrich (Poznan, Poland), while sodium sulphate and sulphuric acid were from Merck (Darmstadt, Germany).

The following analytes were determined: 2,3,7,8-chloro-substituted dibenzo-*p*-dioxins and dibenzo-furans (17 congeners), non-*ortho*-substituted polychlorinated biphenyls, IUPAC numbers 77, 81, 126, and 169 (dL-PCB), and mono-*ortho*-substituted dL-PCBs, IUPAC numbers 105, 114, 118, 123, 156, 157, 167, and 189, and six ndl-PCBs (IUPAC 28, 52, 101, 138, 153, 180). All ^13^C-labelled standards were obtained from Cambridge Isotope Laboratories (Andover, MA, USA) or from the Wellington Laboratories Inc., the ON, Canada, and diluted volumetrically in A-class glass to working concentrations in toluene (PCDD/PCDFs) and isooctane (PCBs).

### 2.3. Sample Processing and Analysis

 Each sample of herring consisted of a combined pooled tissues from five to twelve individual fish, depending on their size. The sample of sprat comprised from thirty to sixty individuals. Salmon, sea trout, and cod were tested individually. Fish muscles homogenate was freeze-dried and extracted by accelerated solvent extraction (ASE 300). The lipid content of the fish sample was determined gravimetrically from the extract. The quantification of the studied compounds was based on the use of ^13^C-labeled internal standards that were spiked into the sample extracts before extraction.

 The analytical method was the same as in our previous published papers [9]. The lipids were decomposed by passing the extract through a multilayer silica gel column eluting with *n*-hexane. The purification and separation was performed on Florisil column by eluting PCBs with *n*-hexane and PCDD/PCDFs with toluene. The fraction containing PCDD/PCDFs was cleaned up on Carbopack C column and diluted with toluene. Separation of mono-*ortho-* PCBs from non-*ortho- *dL-PCBs was achieved by Carbopack C/Florisil column by elution with *n*-hexane and with toluene. Before instrumental analysis, the recovery standards were added. The obtained three sample fractions, containing (1) PCDD/PCDFs, (2) non-*ortho-* PCBs, and (3) mono-*ortho-* PCBs, were all analyzed using HRGC/HRMS.

### 2.4. Instrumental Analysis

 Dioxins and PCBs concentrations were determined by high-resolution gas chromatography coupled to high-resolution mass spectrometry. MAT 95XP (Thermo Scientific, Bremen, Germany) coupled with an Ultra Trace GC (Thermo Scientific, Milan, Italy) with GC PAL autosampler (CTC Analytics AG, Zwingen, Switzerland) was used. Chromatographic separation was achieved by splitless injection of 1 *μ*L on a DB-5MS column (60 m, id 0.25 mm, 0.1 *μ*m, J&W Scientific, Folsom, CA, USA). The HRMS was operated in selective ion monitoring (SIM) mode utilizing resolution of 10, 000. The two most intense ions were monitored for native and labelled compounds. Blank and QC samples were analysed with every batch. Method was validated, and uncertainty of measurement was estimated (14.30% for PCDD/PCDFs, 16.74% for sum of PCDD/PCDF/dL-PCBs, and 22.67% for ndl-PCBs). The limits of detection (LODs) for PCDD/PCDFs and dL-PCBs congeners were isomer dependent and varied between 0.01 and 0.25 pg/g w.w. for PCDD/PCDFs and from 0.5 to 40 pg/g w.w. for PCBs. The recoveries of the internal standards ranged between 60% and 120% for PCDD/PCDFs and 40–150% for PCBs.

### 2.5. Calculations

 Toxic equivalents (TEQs) for PCDD/PCDFs and dL-PCBs were calculated according to toxic equivalency factors (TEFs) adopted by the WHO [15]. The concentrations below LOQs were equated to the LOQ (upperbound concept). These data are expressed as pg WHO-TEQ/g of wet weight (w.w.). Ndl-PCBs concentrations are presented as ng/g of w.w.

### 2.6. Quality Assurance/Quality Control

 All PCDD/PCDFs and PCBs data were assessed for compliance with published acceptance criteria, and the method performance criteria guidelines are laid down in Regulation 1883/2006/EC. The GC-MS analytical run for each set of analyses was preceded by a reference standard solution used to check system performance and calibration validity prior to continuation of the run. The reference standard solution was also analyzed during and at the end of the analytical run. All integrated chromatograms were scrutinized to assess chromatographic peak shape, resolution, and signal-to-noise, and, for high-resolution mass spectrometry, lock-mass traces were examined for evidence of ionization suppression. Isotope ratios for signal peaks were assessed for agreement with theoretical abundances, and the variation in response factors for reference standard solutions within a run was limited to 15%. QA/QC was performed through the analysis of procedural blanks, a duplicate sample (duplicate only for noncompliant samples), and standard reference materials (T620, T637, T645 cod liver oils (FAPAS)) for each set of samples. For the replicate and standard reference materials, the relative standard deviations (RSDs) were <15% for all the detected compounds. Additionally, the method performance was assessed through participation to interlaboratory studies organized by EURL for Dioxins and PCBs in Feed and Food (Freiburg, Germany). 

## 3. Results and Discussion

### 3.1. Concentrations of PCDD/PCDFs and PCBs

**Figure 2:**
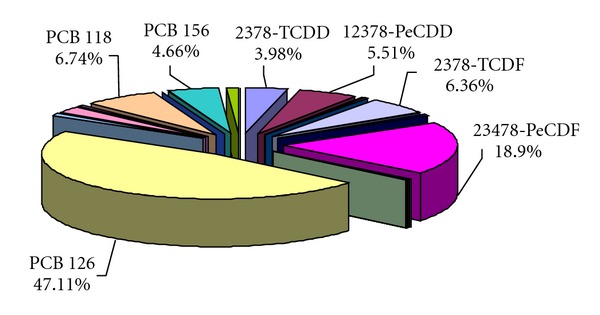
Congener contribution to dioxin-like toxicity in Baltic salmon (*Salmo salar*).

Summaries of chemical analysis of dL-PCBs and PCDD/PCDFs levels in Baltic fish surveyed in 2006–2010 are illustrated in Table 1, while Table 2 shows number of samples that did not meet the requirements of Regulation 1881/2006 or 2006/88/EC. The EU legal limit in fish for the sum of PCDD/PCDFs is 4 pg WHO-TEQ/g wet weight, while for the sum of PCDDs, PCDFs, and dL-PCBs cannot exceed 8 pg WHO-TEQ/g wet weight. Action level is 3 and 6 pg WHO-TEQ/g w.w. for PCDD/PCDFs and dL-PCBs, respectively. Levels of PCDD/PCDFs and PCBs congeners in Baltic fish were stable during the period of 2006–2010 and were different for tested fish species. The highest concentrations of all tested 35 compounds were found in salmon tissues and the lowest in the cod muscles, which contained only 0.4% of fat (Table 1 and Figure 2). The contaminant levels varied among tested fish species, but furans were the dominating compounds in PCDD/PCDFs fraction. PCDD/PCDF/dL-PCBs exceeded the permissible limit in 14 salmon samples and one herring and one sprat sample. PCDD/PCDFs concentration range in noncompliant salmon samples was from 3.10 ± 0.44 to 5.67 ± 0.81 pg WHO-TEQ/g w.w while for the sum of PCDD/PCDF/dL-PCBs was from 9.55 ± 1.54 to 14.11 ± 2.36 pg WHO-TEQ/g w.w. Dioxin-like PCBs accounted for more than 50% in all species of fish.

Indicator PCBs (ndl-PCBs) were significantly below the limit planned by EU for these compounds; these were to be introduced into EU legislation in 2012 (Table 3).

### 3.2. Congener Profiles

 The congener profiles were rather similar among the different species. With regard to PCDD/PCDFs, congener-specific analysis revealed that certain compounds occurred frequently (2,3,7,8-TCDD and 2,3,7,8-TCDF, 1,2,3,7,8-PeCDD, 1,2,3,7,8-PeCDF, 2,3,4,7,8-PeCDF, 1,2,3,4,7,8-HxCDF, 1,2,3,6,7,8-HxCDF, 2,3,4,6,7,8-HxCDF, 1,2,3,6,7,8-HxCDD), others were present only in some fish species (1,2,3,4,7,8-HxCDD, 1,2,3,7,8,9-HxCDD), and the remaining such as 1,2,3,4,6,7,8-HpCDD, OCDD, 1,2,3,7,8,9-HxCDF, 1,2,3,4,6,7,8-HpCDF, 1,2,3,4,7,8,9-HpCDF, and OCDF were not detected in any fish samples. A more detailed examination of results showed that of the 12 dL-PCBs congener peaks for which analyses were conducted in this study (PCBs 77, 126, 169, 105, 114, 118, 123, 156, 157, 167, 189) were detected in all fish; PCB 81 was present in most of the examined samples, excluding the herring samples where PCB 81 was below the limit of detection. The preferential accumulation of congeners in Baltic fish emphasizes the importance of habitat in bioaccumulation of these contaminants. PCB 153 and PCB 138 were the most commonly detected ndl-PCBs in the Baltic fish study. The carried-out examination showed that the sum of the six ndl-PCBs was on average close to five times higher than the sum of the 12 dL-PCBs [2].

### 3.3. Potential of Toxic Congeners

**Figure 3:**
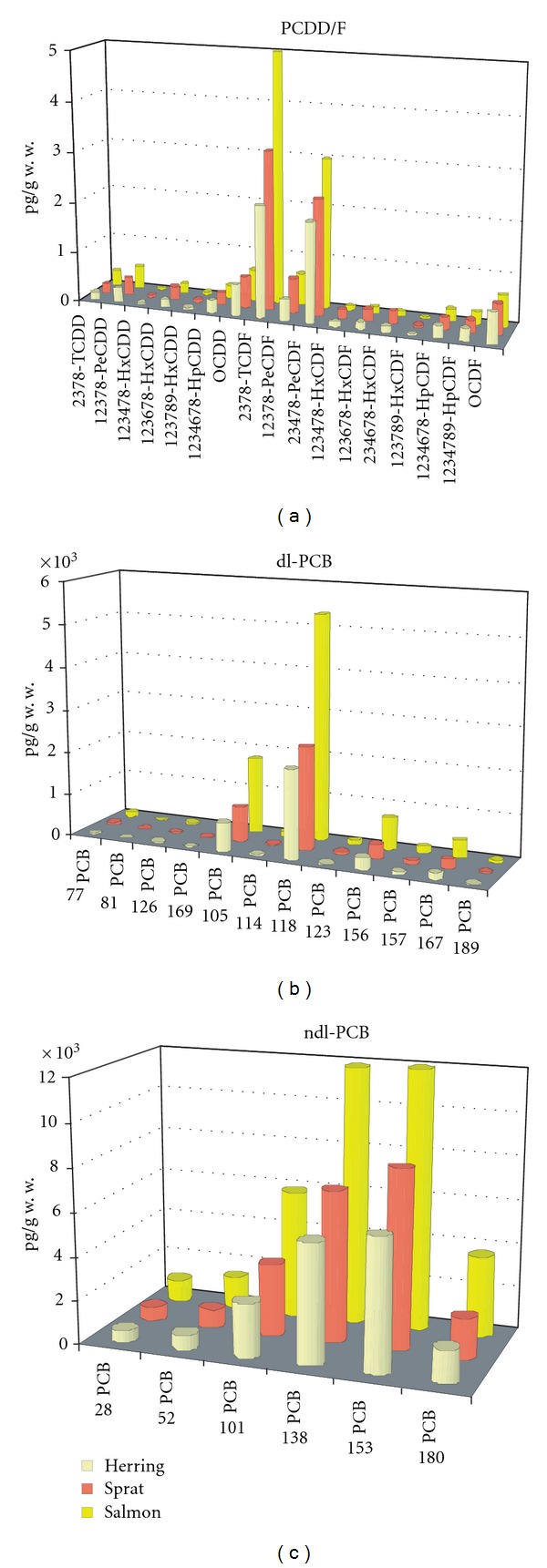
PCDD, PCDF, dL-PCB, and ndl-PCB congener profiles.

 The EU has established maximum limits for these undesirable substances, aiming to ensure that fish is safe for consumer. The received data have showed that the contaminant levels were well below the permit levels in most Baltic fish catches from south-western sea region. By means of the toxicity factors WHO-TEF_1998_, the largest contribution to the toxicity was found to be caused by, successively, PCBs 126, 118, 156, furans (2,3,4,7,8-PeCDF and 2,3,7,8-TCDF), and two dioxins: 1,2,3,7,8-PeCDD and 2,3,7,8-TCDD (Figures 2 and 3). Ndl-PCB contribution to the matrix toxicity was mostly from congener 138, 153, 180, and 101 (Figure 3).

The human dietary intake of dioxin-like PCBs and PCDD/PCDFs from seafood consumption is very different in various countries around the world and largely depends on dietary habits [4–11, 16–18]. In some countries it can reach even more than 50% of the tolerable weekly intake (TWI) set by the Scientific Committee on Food of the European Commission [18]. The level of fish consumption in Poland is among the lowest in the European Union, as indicated by existent studies [19]. Consumption of fish and fish products among adult Poles is about 15-16 g/day/person and is twice lower than recommended. Since fish consumption in Poland is very low, the dioxin dietary intake was much below the TWI set by EC at 14 pg TEQ/kg b.w./week [3]. Thus, consumption of the saltwater fish was not a health risk, although consumption of some of them in large quantities may be harmful to the health of the consumer.

### 3.4. Comparison with Other Countries

 Most Member States of the Baltic Sea coast run numerous programs for the monitoring of dioxins in fish [4–11, 16–18, 20]. Scientific data indicate that dioxin levels in fish depends on many factors, such as a species, fish age, fat content, type of tissues and organs tested, water pollution, fishing area, season, and habit migrations [1, 3, 16, 21]. Some regional differences in organohalogen concentrations are observed. PCDD/PCDFs and PCBs concentrations were significantly higher in the northern than in the southern Baltic Sea fish [4, 5, 7–11]. In some areas, substantial fishing portion of fatty fish, such as herring and salmon, does not correspond with acceptable levels and therefore was excluded from the Swedish and Finnish diet (1881/2006). There is reason to believe that the exclusion of the Baltic fish from the diet may have a negative impact on the health of residents [12]. In these countries, however, the system provides full information to consumers about dietary recommendations in order to avoid the risk in the most vulnerable population groups. In tissues of older Baltic herring, salmon, and some sprat, the dioxins are at levels exceeding the maximum level within the meaning of Regulation 1881/2006/EC. Under European law, maximum limits may not be exceeded in food marketed. Those regulations prohibit the mixing of products complying with the acceptable limits with products exceeding these levels, or the use of noncompliant products as an ingredient in the production of other foodstuffs. In comparison with the results presented by Finland, Sweden, and Germany, the contents of tested compounds presented in the national official surveys from Poland are lower [5, 7, 10, 11, 17, 20].

The results of monitoring of fish in the member countries, covering the period 1999–2008, elaborated recently by EFSA, became the basis for the amendment of Commission Regulation 1881/2006 as regards maximum permitted levels of dioxins and PCBs [1, 2].

## 4. Overall Conclusion

Conducted surveys demonstrate that fatty fish contain higher concentrations of PCDD/PCDFs and PCBs and they mostly exceeded the EU's maximum permissible level or action levels. PCBs congeners including PCBs IUPAC numbers 105, 118, 126, 156 and 101, 138, 153, 180 followed by PCDFs and PCDDs were the dominating pollutants in the examined Baltic fish. The contribution of dL-PCBs to the total dioxin-like toxicity was larger than the contribution of PCDD/PCDFs. Taking into account toxic properties (TEF) of congeners only, two furans, two dioxins, and three congener's dL-PCBs from 29 tested compounds were mostly responsible for the dioxin-like toxicity.

The treaty of Stockholm Convention obliges signatories to take all measures to eliminate (if possible) or reduce (where you cannot eliminate) all sources of dioxins. The immediate objective of the survey was therefore to obtain information about existing levels of pollutants, taking preventive actions and assessing risk. Although the dietary consumption of fish from southwest region of Baltic Sea did not represent a risk for human health (because of low consumption of marine fish), the excessive eating of some of them may be of significance for health of various subgroups of consumers (fishermen). There is no chance of removing dioxin and related pollutants from the sea. Since the level of fish contamination is dependent on the aquatic environment, human exposure can only be reduced through more effective fish control.

## Figures and Tables

**Table 1:** PCDD/PCDFs and dL-PCBs in Polish Baltic fishing area. Average and range concentration (pg WHO-TEQ/g w.w.).

Fish species	Year	pg WHO-TEQ/g w.w						
		PCDD/F $\overset{®}{x}\pm std.dev$.	dL-PCB $\overset{®}{x}\pm std.dev$.	*∑* PCDD/F/dL-PCB $\overset{®}{x}\pm std.dev$.	Min	Max	Min	Max
					PCDD/F *x* ± U*		*∑* PCDD/F/dL-PCB *x* ± U*	
Salmon *n* = 52	2006 2007 2008 2009 2010	3.42 ± 1.13 2.87 ± 0.99 2.57 ± 1.17 2.75 ± 0.84 3.04 ± 0.73	5.81 ± 1.53 4.83 ± 1.43 4.78 ± 1.93 5.12 ± 1.41 5.31 ± 1.33	9.23 ± 2.64 7.70 ± 2.39 7.35 ± 3.07 7.87 ± 2.18 8.35 ± 2.00	0.64 ± 0.09	5.67 ± 0.81	1.69 ± 0.28	14.11 ± 2.36

Herring *n* = 52	2006 2007 2008 2009 2010	2.15 ± 0.61 1.87 ± 1.06 1.52 ± 0.44 1.32 ± 0.36 1.55 ± 0.68	2.29 ± 0.75 2.09 ± 0.91 1.74 ± 0.44 1.31 ± 0.35 1.60 ± 0.57	4.45 ± 1.35 3.96 ± 1.96 3.26 ± 0.87 2.63 ± 0.62 3.15 ± 1.22	0.63 ± 0.09	4.64 ± 0.66	1.36 ± 0.23	9.07 ± 1.52

Sprat *n* = 52	2006 2007 2008 2009 2010	2.71 ± 0.51 2.11 ± 0.84 2.10 ± 0.26 1.66 ± 0.98 1.94 ± 0.73	3.33 ± 0.56 2.85 ± 0.70 2.74 ± 0.2 2.34 ± 0.91 2.39 ± 0.65	6.06 ± 1.01 4.96 ± 1.48 4.84 ± 0.41 4.17 ± 1.68 4.33 ± 1.36	0.23 ± 0.03	3.88 ± 0.56	0.85 ± 0.14	8.01 ± 1.34

Sea trout *n* = 6	2009 2010	3.07 ± 1.25 2.99 ± 0.04	6.07 ± 2.56 5.00 ± 0.48	9.14 ± 3.78 7.99 ± 0.51	1.93 ± 0.28	4.40 ± 0.63	6.13 ± 1.02	13.38 ± 2.24

Cod *n* = 15	2009 2010	0.22 ± 0.00 0.22 ± 0.01	0.64 ± 0.02 0.64 ± 0.04	0.86 ± 0.03 0.85 ± 0.02	0.22 ± 0.03	0.23 ± 0.03	0.84 ± 0.14	0.90 ± 0.15

U*: expanded uncertainty (Eurachem/Citac Guide CG4 “Quantifying Uncertainty in Analytical Measurements”).

**Table 2:** Number of samples noncompliant with maximum or action level for PCDD/PCDFs and sum of PCDD/PCDF/dL-PCB (1881/2006/EC and 2006/88/EC).

Fish species	No. of samples analyzed	PCDD/PCDF maximum/action level	*∑* PCDD/PCDF/dL-PCB maximum level	dL-PCB action level
Salmon	52	8/3	14	44
Herring	52	0/1	1	2
Sprat	52	0/1	1	5
Sea trout	6	0/1	1	6
Cod	15	0/0	0	0

**Table 3:** Marker PCBs in Polish Baltic fishing areas (ng/g w.w.).

Fish species	Concentration range (ng/g w.w.)		Mean (ng/g w.w.)
	Min	Max	
Salmon *n* = 52	7.72 ± 1.75	60.84 ± 13.79	36.20 ± 11.62
Herring *n* = 52	6.47 ± 1.47	42.16 ± 9.56	16.29 ± 6.68
Sprat *n* = 52	1.46 ± 0.33	46.02 ± 10.43	20,78 ± 8.14
Sea trout *n* = 6	30.77 ± 6.98	56.43 ± 12.79	38.66 ± 9.20
Cod *n* = 15	0.07 ± 0.02	2.78 ± 0.63	1.11 ± 0.68
